# Mechanics of proteins with a focus on atomic force microscopy

**DOI:** 10.1186/1477-3155-11-S1-S3

**Published:** 2013-12-10

**Authors:** Felix Rico, Annafrancesca Rigato, Laura Picas, Simon Scheuring

**Affiliations:** 1 U1006 INSERM, Aix-Marseille Université, Parc Scientifique de Luminy, 13009 Marseille, France; 2 Institut Curie, UMR144 CNRS, 26 rue d'Ulm, 75005 Paris, France

## Abstract

The capacity of proteins to function relies on a balance between molecular stability to maintain their folded state and structural flexibility allowing conformational changes related to biological function. Among many others, four different examples can be chosen. The giant protein titin is stretched and can unfold during muscle contraction providing passive elasticity to muscle tissue; myoglobin adsorbs and releases oxygen molecules thank to conformational changes in its structure; the outer membrane protein G (OmpG) is a bacterial porin with a long and flexible loop that modulates gating; and the proton pump bacteriorhodopsin adapts its cytosolic half to allow proton pumping. All these conformational changes triggered either by chemical or by physical cues, require mechanical flexibility or elasticity of certain protein domains. While the methods to determine protein structure, X-ray crystallography above all, have been dramatically improved over the last decades, the number of tools that directly measure the mechanical flexibility of proteins and protein domains is still limited. In this tutorial, after a brief introduction to protein structure, we present some of the available techniques to estimate protein flexibility, then focusing on atomic force microscopy (AFM). We describe the principles of the technique and its various imaging and force spectroscopy modes of operation that allow probing the elasticity of proteins, protein domains and their surrounding environment.

## Introduction

Proteins are essential building blocks of living organisms and function as bricks, scaffolds, channels, nutrient transporters and force transducers, working as the robots of Nature. Similar to architectural structures, which are built into a variety of shapes and geometries suitable for sustaining moderate or important loads, the sequences of amino acids that form proteins fold into different structures that can vary in flexibility, allowing or preventing conformational changes related to function [1]. Like all architectural arches, made of the very defined materials, such as brick and concrete, proteins are made of the 20 known amino acids with identical backbone. However, even if the mechanical properties of concrete and brick remain the same, some structures support load more efficiently than others, such as catenary arches used in Gaudi architecture compared to Roman ones. Similarly, some structural motifs of proteins may be rigid providing structural stability, while others may be flexible allowing conformational changes. In this tutorial, we will describe different methods to determine the rigidity, elasticity or flexibility of proteins and protein motifs. Since the final flexibility of proteins mainly depends on the structural conformation adopted by the chain of amino acids, we start with a brief introduction to protein structure.

### Protein structure

**Figure 1:**
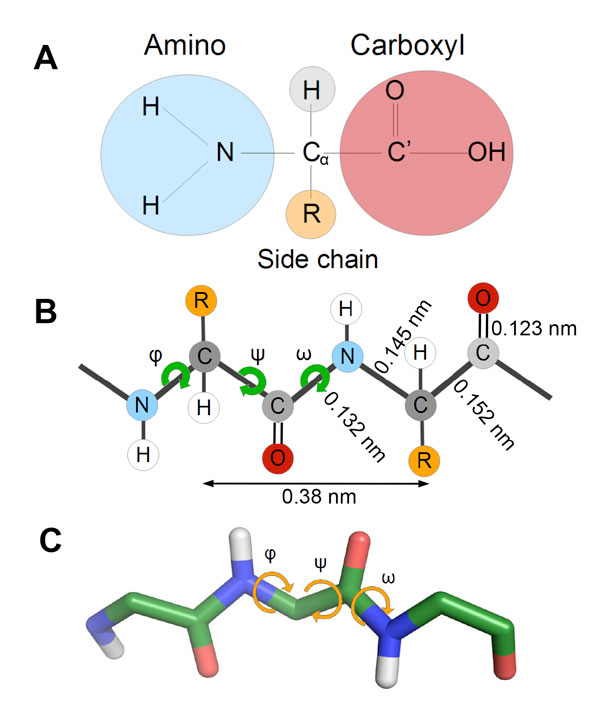
**Proteins are chains of amino acids**. A) Structure of a typical amino acid, showing the central C_α _atom with a hydrogen atom attached to it, an amino group (NH_2_), a carboxyl group (COOH, carbon atom named C') and the side chain (R) that varies among the different amino acids. B) Schematic representation of a polypeptide chain with two amino acids, showing average distances of bonds. The main chain atoms between two C_α _(dark gray) atoms are generally fixed in a plane, with the C'-N peptide bond angle (ω) being 0º or 180º (trans, most common, or cis forms), thus only the C_α_-C' and N-C_α _bonds exhibit rotational mobility. C) Polypeptide chain formed by three glycines showing the bond angles. C atoms in green, O in red, N in blue and H in white. As the C_α _is always saturated, the two H linked to it are omitted.

Proteins are made of one or more linear chains of amino acids (aa) linked to one another by peptide bonds. When chains have a small number of aas they are named peptides (<50 aas). Thus, proteins are linear polypeptide chains. Amino acids have a common structure consisting of a central carbon atom (named C_α_) with a hydrogen atom attached to it, an amino group (NH_2_) and a carboxyl group (COOH, whose carbon atom is named C'). The side chain (R) attached to the C_α _atom is variable and provides the identity to each amino acid (Figure 1A). There are 20 different amino acids in natural proteins, each one with a different side chain and a common entity (NH-C_α_H-C' = O). During protein synthesis, amino acids are covalently linked by so-called peptide bonds, that form between the carboxyl group of one aa (*n*) with the amino group of the following one (*n*+1) leaving a molecule of H_2_O as by product (one H from the amino group and one O and one H from the carboxyl group). Amino acids are then named residues. Thus, the first amino and the last carboxyl groups remain intact and determines the order of the chain, which extends from the amino (N) terminus to the carboxyl (C) terminus. The main chain or backbone of a protein is therefore formed by a series of residues joined by peptide bonds and decorated by the different side chains (Figure 1B). The number of aas of a protein varies from a few tens (*e.g*. ~50 for insulin) to over several tens of thousands (*e.g*. ~30.000 for giant muscle protein titin).

As mentioned above, what differs from one aa to another is the side chain. Thus, the side chain provides the specific properties to each aa that can be charged, uncharged or hydrophobic. Three amino acids differ from the others for the unique properties of their side chains: glycine (G, Gly, amino acids can be named by single or three-letter codes.), with a single H atom as side chain; proline (P, Pro), whose side chain forms a cyclic pyrrolidine ring from the C_α _atom but linked to the amino group; and cysteine (C, Cys), that has a thiol at the end of its side chain able to form disulfide bonds, which can confer stability to some proteins and/or rigidify the structure of certain domains (for a full list of known amino acids, please refer to textbooks, such as Branden and Tooze [2]).

Although the primary structure (the sequence of aas) provides important information about the protein, the functionality of a protein mainly relies on the three-dimensional fold adopted by the polypeptide chain. Therefore, to allow the protein to fold into its final structure, bonds between backbone atoms require certain mobility. Only the two dihedral angles of the C_α_-C' and N-C_α _bonds, named respectively psi (ψ) and phi (ϕ), exhibit rotational mobility and define the degrees of freedom that determine the conformation of the peptide chain, *i.e*. the structure of the protein (Figure 1B and 1C). Knowing the pair of angles for each peptide unit within a protein is enough to determine its structure.

To preserve the particular angles that determine the conformation of the peptide chain and the maintenance of the final protein structure, inter- and intra-peptide forces are required. These forces mainly involve hydrogen bonding, electrostatic and van der Waals interactions and, importantly, attractive interactions arising from the hydrophobic effect (less well characterized). All these forces have in common that they are weak compared to covalent bonds, which already implies a certain flexibility of the protein structure. The quasi-unique combination of these non-specific, inter and intra-peptide interactions lead to the specific and particular conformation of proteins. Among the extraordinary variety of protein structures found in nature, certain structural motifs are frequently observed and characterize the secondary structure of a protein. Given the polar, hydrophilic nature of the main chain, NH being a hydrogen bond donor and C' = O a hydrogen bond acceptor, in some cases consecutive residues have all the same pair of angles (ϕ, ψ) adopting a particular secondary structure that neutralizes the polar groups.

**Figure 2:**
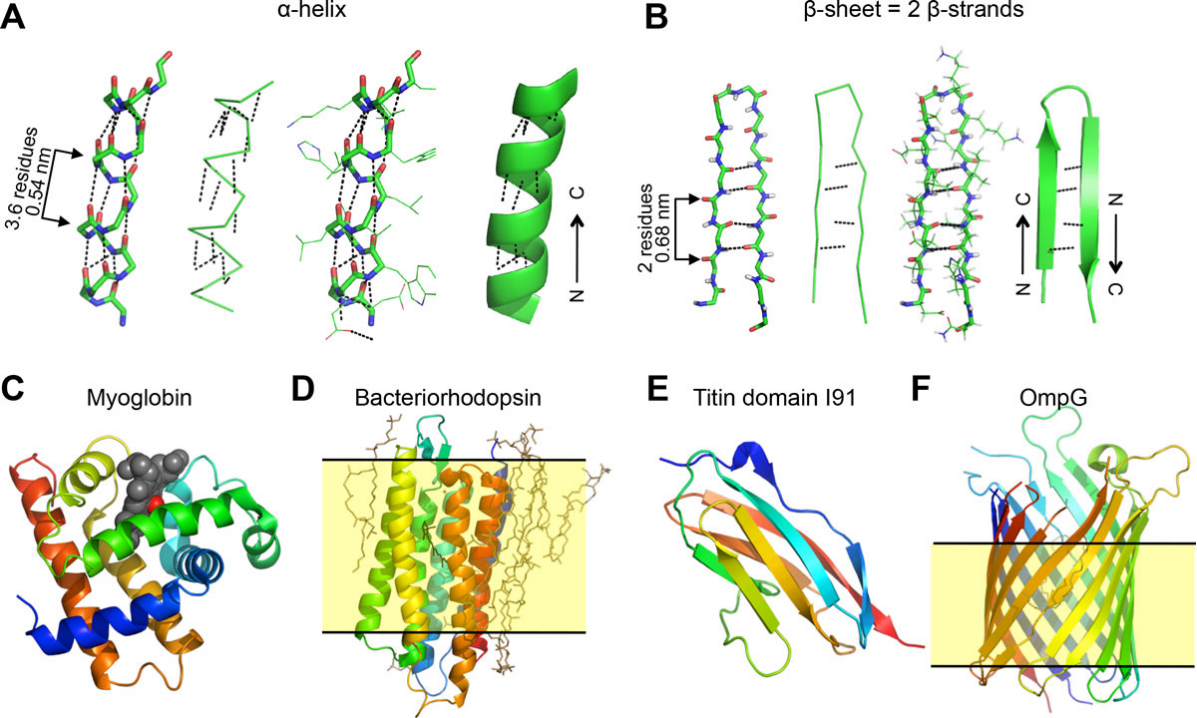
**Secondary structure of proteins**. A) Stick, ribbon, stick with line side chains and cartoon representations of a same α-helix. Dotted lines represent hydrogen bonds between the C' = O of residue *n *and the NH of residue *n*+4, each consecutive residue with approximate angles (-57º, -47º). The helical structure is formed by 3.6 residues per turn and a pitch of ~0.54 nm. B) Stick, ribbon, stick with line side chains and cartoon representations of a same antiparallel β-sheet, made of two β-strands. β-sheets are held together by hydrogen bonds between a C' = O residue of one β-strand and the NH residue of the apposing β-strand. Although very elongated, β-strands are also helical arrangements with two residues per turn and a pitch of ~0.7 nm. C) Cartoon representation of the protein myoglobin (PDB file 1MBN), mainly made of α-helices, showing the heme group as gray spheres and the oxygen molecule in red. It has a size of 2 × 3 × 4 nm^3^. D) Cartoon representation of membrane protein bacteriorhodopsin (PDB file 2at9), revealing the seven transmembrane α-helices, lipid molecules as brown sticks and the retinal in the protein core as black sticks. The yellow region represents the lipid bilayer with a typical thicknes of ~5 nm. E) Cartoon representation of muscle protein titin domain I91 (former I27) mainly formed by β-strands (PDB file 1TIU). F) Cartoon representation of outer membrane protein G, revealing its β-barrel conformation formed by 14 β-strands (PDB file 2F1C). The yellow region represents the lipid bilayer. The colour scale of images C to F goes from the N-terminus (blue) to the C-terminus (red), not to be confused with B-factors colour scale shown in Fig. 3B.

This is the case for the two most common secondary structure motifs: α-helices and β-sheets (Figure 2). Some particular motifs forming loops have also been identified, but appear to be less structured. Figure 2A shows different representations of a same α-helix, the most common motif in proteins, held together by a unique network of hydrogen bonds. α-helices were initially predicted by Linus Pauling, and the first experimental evidence of their existence was provided by the diffraction pattern of hemoglobin. Myoglobin, shown in Figure 2C, was the first protein to have its three-dimensional structure solved (Perutz and Kendrew, Nobel Prize for Chemistry 1962), revealing eight right-handed α-helices (Figure 2C). β-sheets are formed by two parallel β-strands in a parallel (-139º, +135º) or antiparallel (-119º, +113º) configuration that are held together by hydrogen bonds (Figure 2B). An example of a protein structure formed mainly by β-strands is found in the immunoglobulin domains of titin (Figure 2E). Loop regions commonly connect α-helices and/or β-strands within proteins, such as β-hairpin loops connecting two adjacent β-strands (Figure 2B), and tend to be less structured and more flexible. The number of residues forming a loop commonly varies between 2 and 6, although some loops can be formed by more than 20 residues [3].

Finally, the tertiary structure of proteins is what defines the overall shape of a single protein and thus, what controls the basic function of a protein. The tertiary structure describes the spatial interaction between secondary structures and is commonly due to hydrophobic interactions, disulphide bonds and salt bridges.

Folded proteins can also be classified based on the way they interact with lipid membranes giving rise to three different families of membrane proteins (besides the soluble proteins): integral membrane proteins, lipid-anchored proteins and peripheral membrane proteins. Unlike other soluble proteins that expose most of their surface to an aqueous environment, integral membrane proteins reside on a lipid bilayer, exposing most of their surface to a hydrophobic environment. Although most membrane proteins are formed by α-helical structures, like bacteriorhodopsin (bR) (Figure 2D), β-strand rich membrane proteins exist that are sufficiently hydrophobic to reside within membranes, such as OmpG (Figure 2F). The difficulty of studying membrane proteins resides in their requirement to reside in a hydrophobic lipid microenvironment, which is often difficult to mimic during or even after protein purification.

Despite decades of efforts towards understanding how proteins fold, the general mechanism by which a linear sequence of amino acids folds into a "unique" three-dimensional structure is still an unanswered question in Biology. An early assumption to the folding mechanism was that the polypeptide chain explored all the possible conformations until the minimum energy of the native state is found. In the late 1960's, Cyrus Levinthal showed that this hypothesis was impossible through a simple calculation of the time required to complete this process[4]. For a protein of 100 aas, assuming that each peptide has only three possible conformations, the polypeptide chain will have 3^100 ^= 5 × 10^47 ^possible conformations. Assuming the shortest time of 1 picosecond (10^-12 ^s) to change from one conformation to another, the protein would require ~10^28 ^years to find its native state, while proteins are known to fold in a much shorter time, from a few milliseconds to some minutes[2]. Thus, it seems that proteins do not use this mechanism for folding. Although some possible explanations have been proposed, Levinthal's paradox still remains an open question in the field of protein folding.

## Flexibility of proteins

The protein structures shown in Figure 2C-F appear as rigid, static elements. However, proteins are dynamic and constantly subjected to random perturbations coming from the aqueous and/or lipid environment. In fact, due to thermal energy (*k*_B_*T*~4.1 pN/nm or ~0.592 kcal/mol, *k*_B _being the Boltzmann constant and *T*, the absolute temperature) proteins constantly fluctuate from the minimum of their conformational free energy landscape [5]. A well-studied example is the above-mentioned myoglobin, a protein found in many organisms whose function is to store, transport and liberate oxygen in muscles. The heme group to which oxygen adsorbs in myoglobin is partially buried inside the protein core. Hence, myoglobin requires conformational changes to adsorb and then liberate oxygen molecules. Indeed, these motions have been observed in temperature dependent X-ray diffraction studies and led to the concept of roughness of the free energy landscape. Consequently, it is now accepted that proteins have a complex, rugged folding energy landscape with a number of local minima from and to which the protein hops constantly at physiological temperatures and that are essential for biological function[6].

The second example of protein structure shown in Figure 2E is the immunoglobulin-like domain I91 of titin. Titin is a giant muscle protein made up of more than 200 domains that provide passive elasticity to muscles and can deform and even unfold during muscle contraction when tension is generated[7]. Thus, it is obvious that the flexibility and mechanical strength of titin domains is crucial for its function, as titin is constantly subjected to mechanical forces.

One of the first solved structures of an integral membrane protein was that of bacteriorhodopsin (Figure 2D), a proton pump that naturally forms 2D crystals in the membrane of a microorganism found in the Dead Sea, *Halobacterium salinarum*. It is mainly composed of seven α-helices rich in hydrophobic residues that align along the plane normal to the membrane and are thus named transmembrane helices. The retinal group buried inside the protein core is responsible of absorbing green light, which provides the characteristic purple colour to isolated membrane fragments (the cells). Upon absorption of a photon, the cytosolic half of the protein changes conformation allowing a proton to diffuse from the cytosolic to the extracellular side. This conformational change again requires certain reversible flexibility of protein domains.

Finally, outer membrane protein G (OmpG, Figure 2F) is a porin found in the outer membrane of bacteria that mediates the non-specific uptake of molecules to the periplasm. OmpG is formed by 14 β-strands forming a structure known as β-barrel. Interestingly, the longest loop 6 is thought to be responsible for the pH-depending gating activity by folding into the barrel lumen, involving the unzipping of hydrogen bonds between two β-strands[8]. Thus, for functioning, OmpG also requires conformational changes, and therefore, certain flexibility. These four examples already justify the necessity of determining the flexibility of proteins, since proteins deform continuously due to thermal motion and adopt different conformations to undergo biological function. We present here an incomplete list of available approaches that lead to an estimation of protein flexibility.

### Ramachandran plots

**Figure 3:**
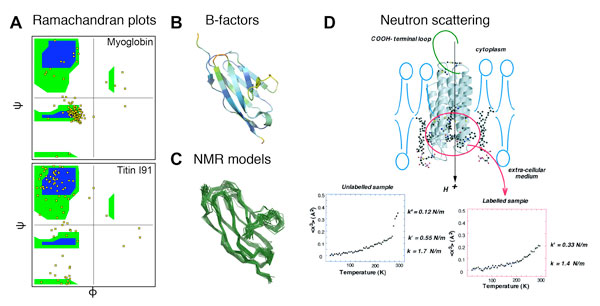
**Methods to estimate protein flexibility**. A) Ramachandran plots of ψ versus ϕ of all amino acids (yellow dots) for myoglobin (top) and titin I91 domain (bottom). The blue regions of the plot are the allowed pairs of angles since they don't present steric repulsion, while green regions present more repulsion and are termed partially allowed regions. B) Cartoon representation of titin domain I91 coloured by B-factors, going from blue representing low B-factors to red for high B-factors. Loops connecting β-strands and N- and C- terminal domains appear in general more flexible than β-sheets. C) Ribbon representation of the family of structures of titin I91 domain obtained by NMR spectroscopy (PDB file 1TIU). The 24 different models calculated from NMR data represent I91 domain conformations that are compatible with the experimental data. They present good superpositions of the regions in beta-sheet fold, while the loops in between and the N- and C-terminal domains show variable positions. These observations are in agreement with the B-factors calculated from X-ray crystallography (Fig. 3B), which are low for β-sheets and higher for the loops and terminals and thus intrinsically more flexible. D) Neutron scattering experiments on bacteriorhodopsin (cartoon) providing the root-mean square displacement of protein atoms (<x^2^>) as a function of temperature. The slope of the curves at physiological temperatures (steeper slope) provided a quantification of the force constant of 0.12 N/m for the whole protein (unlabelled) and of 0.33 N/m for the labelled extracellular half of bacteriorhodopsin (labelled). Reproduced with permission from ref. [9]. Zaccai "From [Full Reference Citation]. Reprinted with permission from AAAS." DOI: 10.1126/science.288.5471.1604

As mentioned before, the conformation of the polypeptide chain of a protein can be determined by knowing the ϕ and ψ angles of each aa, which are included in the solved protein structure. Since each aa has a particular side chain, there are specific pairs of angles that are not allowed because of steric collisions with the main chain (steric hindrance). The aa that can adopt the highest number of different conformations (pairs of angles) is glycine, because it has the smallest side chain (only one H). All possible pairs of angles for the different combinations of aas in a protein can be calculated and represented in the so-called Ramachandran plots (Figure 3A), which represents ψ versus ϕ, each dot representing one aa. Interestingly, most α-helices aas fall around the lower left green/blue region, as reflects myoglobin's data (Figure 3A, top), while β-strands have a wider number of allowed angle pairs, falling near the top left region (as reflects titin I91's data, Figure 3A, bottom). Amino acids falling far from these allowed regions may thus be loop aas or relatively unstable aas, which determine their conformation and, to a certain extent, their flexibility. However, Ramachandran diagrams are in general used to find conformationally unrealistic regions from a particular structure model, since in well-refined models only glycines lie outside of allowed regions.

### B-factors

Most of the solved structures of proteins are determined by X-ray crystallographic studies, which involve obtaining protein crystals that diffract properly. The diffraction pattern obtained from X-ray analysis is then used to calculate the electron density map of a protein, from which its crystal structure is obtained. Temperature, Debye-Waller or B-factors are a measure of the uncertainty in the determination of the atom position in the crystal structure and are defined by

$B_{i}=8\pi^{2}{U_{i}}^{2}$

where *U*_i_^2 ^is the mean square displacement of atom *i *and has units of Å^2^. Thus, B-factors also indicate the thermal motion of each atom. The higher the B-factor, the more the atom moves around its average position, *i.e*., the wider the spreading of its electron density. Although a quantified parameter, B-factors obtained from different techniques or from different proteins are not comparable. Even more, B-factors from a same protein obtained from different crystals are not comparable. Thus, B-factor values are limited to a particular crystal structure, as they depend on the contacts within the crystal, the packing of the crystal and/or the mobility of the atoms within the protein itself. Therefore, B-factors reflect a mix of real displacements due to thermal motion, due to different "stable" conformational states (*e.g*. open and close conformations of a channel) and average displacements about the resting position (dynamic disorder), but always within the restricted mobility of the crystal. For example, it is expected that loosely packed protein crystals may lead to structural domains with high B-factors. Nevertheless, B-factors are useful to estimate the flexibility of atoms or groups of atoms within a protein. Each atom of a protein structure dataset, in addition to its structural information (its position), has an associated B-factor. Typically, the end of chains and loops are more mobile and flexible, thus tend to have high B-factors, while atoms inside tightly packed motifs have lower ones (as reflected in titin domain I91 in Figure 3B). Hence, although the B-factor is a useful parameter to determine the flexibility of protein domains, care should be taken to avoid over interpretation.

### Nuclear magnetic resonance

Nuclear magnetic resonance (NMR) spectroscopy is a powerful technique for the study of protein structure, folding, dynamics and interactions with other molecules. Its major advantage, compared to X-ray diffraction, is that proteins are in solution, thus they do not need to be crystallized. Major drawbacks are that NMR is not suitable for proteins larger than 35 kDa and difficult to apply to membrane proteins, since during purification they require being solubilized with detergents that may compromise their structure (and add to their overall size).

NMR spectroscopy relies on the property of some nuclei to emit radiation when they are placed in a strong magnetic field and perturbed by a radiofrequency pulse. The radiation emitted by a nucleus has a typical resonance frequency that depends on the strength of the external magnetic field, its magnetogyric ratio and its local environment. NMR spectroscopy exploits such dependence on the chemical environment, thus on the position of an atom within the molecule, to obtain structural information. In particular, to calculate the structure of a protein, one measures the resonance frequency of the hydrogen, carbon and nitrogen atoms of each amino acid in the sequence. The correlation between atoms linked by covalent bonds and the distances between atoms which are close in space but not in the primary sequence, are obtained from their dipolar couplings. Thus, 2D spectra provide the distance between all atoms in the protein. A computer algorithm then generates a family of structures, which represent the possible conformations compatible with the experimental data. By comparing the calculated models, one can observe that some regions of a protein are well superimposed in all the structures, while others show significant position variations (Figure 3C). Such differences are precious information regarding the different conformations that a domain can assume and can also provide, in addition, interesting functional clues. For example, if the family of structures obtained presents two well structured domains but only one at a time can be well superimposed in all the models, that means that the linker between the two is very flexible, thus allowing different relative orientations of the two domains. Such a result is typical, for instance, of proteins that regulate the access to their binding site by switching from an open to a closed conformation. Advanced NMR techniques allow determining the relaxation times of the different conformational states and the flexibility of the entire peptide chain, thus revealing regions implicated in structural changes.

### Elastic neutron scattering

The above-described methods to evaluate protein flexibility are qualitative and related to conformational disorder. However, efforts have been made towards a quantification of protein mechanics. An elegant method proposed by Prof. Zaccai suggests using quantitative measurements of thermal fluctuations (<*x*^2^>) as a function of absolute temperature (*T*) from elastic incoherent neutron-scattering experiments (Figure 3D)[9]. At very low temperatures, proteins are "frozen" in a particular conformational sub-state, and vibrate harmonically around the equilibrium sub-state dominated by quantum effects. As temperature increases, vibrations around this sub-state become more important, the mean square displacement increasing linearly with absolute temperature. From this slope of the data, an average force constant (*k*) can be determined. Above a certain temperature, the dynamical transition, thermal energy is high enough to allow the protein to cross barriers between sub-states, thus hopping from one substate to another and vibration becomes anharmonic. This temperature regime reflects the physiological dynamics of the protein and it is thus related to biological function. Although anharmonic motion does not allow a strict definition of an average force constant, a pseudo-force constant can be extracted from the plot reflecting the stiffness or "softness" of the protein

$$<k>=k_{B}/\left(d<3x^{2}>/dT\right).$$

Using this approach, an average spring constant of myoglobin at physiological temperatures was calculated to be ~0.3 N/m. In an effort to get information about the different domains of a protein, using native and deuterated samples of bacteriorhodopsin (bR) it was shown that, the active core of bR was stiffer (0.3 N/m) than the overall protein (0.1 N/m), perhaps reflecting the compact α-helical structure and the higher flexibility of inter-helical loops. Compared to X-ray crystallography, elastic neutron scattering experiments can be carried out on non-crystallized protein, thus minimizing packing and contacts with neighbour proteins. The main advantage of this method is that it provides a quantitative determination of protein elasticity, which allows comparing the flexibility between proteins, although it is restricted to average values or limited to specific tagged domains.

## Direct mechanical measurements on proteins

We have seen that the conventional ways to determine the flexibility or elasticity of proteins are, either indirect, depend strongly on the refinement of the structure or on the crystal packing, or are difficult to apply to proteins in their native environment, such as the lipid membrane. Thus, except for neutron scattering experiments, flexibility estimations are more qualitative than quantitative.

In mechanical engineering, the flexibility of a material is defined by its *elasticity *and is commonly measured by applying a controlled force (*F*, measured in newton, N) or stress (*σ *= *F*/area, measured in pascal, N/m^2^) and measuring the resulting deformation (*δ*, measured in meters) or strain (*ε *= deformation/undeformed length, no units). This leads to a stress/strain relationship, *σ *= *E•ε *that leads to the definition the Young's modulus of elasticity (this relationship is generalization of Hooke's law, *i.e. F *= *k•δ *being *k *the spring constant) [10,11]. Although proteins are not continuous systems, the Young's modulus might not be the most suitable parameter to describe protein flexibility, but a similar procedure might be used. Ideally, to determine the flexibility of a protein, it would be required to apply a controlled force to a certain domain and measure the resulting deformation. However, given the reduced dimension of proteins it is difficult to apply this type of direct mechanical measurement and protein elasticity or flexibility is commonly characterized by indirect methods, as described above. Recently developed methods using nanotechnologies allow now direct mechanical measurements on individual proteins. The emergence of nanotools such as optical tweezers or atomic force microscopy (AFM) allowed the mechanical measurement of individual proteins with nm resolution and the application of forces in the pN regime. Although we will focus on mechanical measurements with AFM, the type of experiments and the resulting data are in general very similar between the two mentioned techniques.

**Figure 4:**
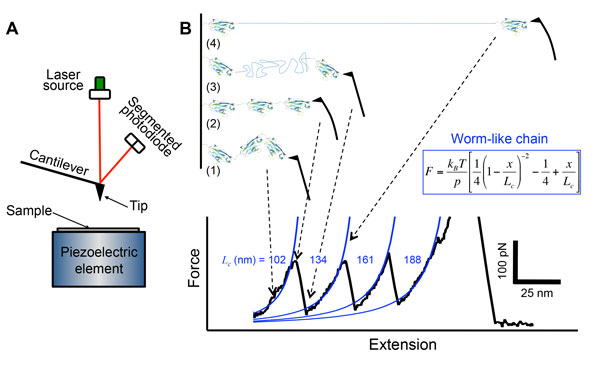
**Atomic force microscopy**. A) Basic components of an atomic force microscope: a laser beam is reflected off the back side of a cantilever with a sharp tip at its end. The reflected light is detected by a segmented photodiode to measure the cantilever deflection. The sample stage is positioned relative to the tip with subnanometer resolution in the three dimensions of space using piezoelectric elements. B) Protein unfolding using force spectroscopy. The plot shows a force versus extension curve of the pulling process of a multimer of titin I91 domains with an AFM tip (black solid line). The initial regime reflects the stretching of the multimer (1), followed by the unfolding of one domain (2) characterized by a drop in force (3). The forced extension of the unfolded polypeptide chain (4) can be explained by the worm-like chain model (blue solid lines). Notice that unfolding forces are slightly different between each other even if the unfolded domain is in all case the I91 domain.

Atomic force microscopy (AFM) is a versatile tool that allows imaging the sample surface with nanometer resolution. An atomic force microscope uses a flexible cantilever as a force probe. An optical system composed of a light source and a segmented photodiode, measures the deflection (*d*) by focusing a laser beam on the backside of the cantilever and detecting the reflected light with the photodiode (Figure 4A). The cantilever behaves like a spring, thus force (*F*) is proportional to deflection *F = k•*, with *k *the spring constant of the cantilever, commonly of 10-100 pN/nm for biological applications. The cantilever has a sharp tip at its end that makes actual contact with the sample surface and it is positioned relative to the sample using piezoelectric elements that allow precise control in the three dimensions of space. Although initially designed as an imaging tool, AFM can also be used for measuring or applying mechanical forces ranging from 10 pN to 100 nN. In force spectroscopy mode, the piezo stage is moved in the z-direction providing series of force versus distance curves that allow determining the mechanics of a sample in compression or under tension.

### Force spectroscopy using AFM

After the first measurements of the binding strength of single receptor/ligand complexes using AFM[12-14], researchers studied the mechanical behaviour of multidomain proteins such as titin by applying tension on the N- and C-terminus using optical tweezers and AFM[15,16]. Unlike linear, elastic materials in which an applied force leads to a proportional deformation, titin revealed a "now" characteristic saw-tooth like pattern in which each peak corresponds to the unfolding of a protein domain (Figure 4B). Thus, most of titin protein domains appear to unfold in a two-state process, from the folded to the unfolded state. Intermediate states were later described from measurements using multimers of a same titin domain, reflecting a more complex behaviour[17]. Thus, force spectroscopy allows measuring the force at which a protein unfolds, a parameter conceptually similar to the tensile strength of a material, *i.e*. the force at which a material breaks or fails to behave elastically. However, given the reduced dimensions of proteins, they are constantly being perturbed by thermal fluctuations and unfolding is therefore a stochastic process that leads to a distribution of unfolding forces and not to a deterministic value. In addition, since unfolding is an activated process that involves crossing an energy barrier helped by the applied force and the thermal bath, unfolding forces depend also on the velocity of pulling. At the pulling rates accessible to AFM, the process still requires some thermal energy to help it crossing the barrier. Thus, the slower we pull, the more time we give to the bath to provide this additional energy and the protein unfolds at low forces. Inversely, if we pull fast, shorter time is given to the bath to provide this energy, thus the protein unfolds at higher forces. Notice that, at the nanoscale, the theoretical interpretation of mechanical measurements is in general different than at the macroscale, and new theories have emerged[18].

In addition to the mechanical resistance upon unfolding, force spectroscopy also provides a measure of the elasticity of the unfolded polypeptide chain. The shape of the curve before rupture occurs is noteworthy (step 3 in Figure 4), it represents the forced extension of the unfolded chain. It can be described using the worm-like chain (WLC) model for polymer stretching[19]. Although this is a continuous model for a chain that requires an energetic cost to bend, it can be discretized into a chain of domains with identical harmonic bending potential for each pair of domains. The WLC model describes the force as a function of the extension (*x*), the contour length (*L_c_*) and the persistence length (*p*) of each domain

$$F=\frac{k_{B}T}{p}\left[\frac{1}{4}{\left(1-\frac{x}{L_{c}}\right)}^{-2}-\frac{1}{4}+\frac{x}{L_{c}}\right]$$

For proteins, the persistence length is commonly set to 0.38 nm, that corresponds to the full extended length of an amino acid backbone (see above), and reflects the rigidity of the chain units. As shown in Figure 4 for titin I91 domain and as described by the WLC model, the force increases nonlinearly before each unfolding event, tending to infinity when the contour length is reached. This force regime corresponds to the stretching of the multimer before the first unfolding peak, and to the stretching of the unfolded polypeptide chain after unfolding occurs. The distance between peaks reflects the length of the unfolded and stretched polypeptide chain of each domain minus the folded length of the domain (~4 nm for titin I91). Thus, after each unfolding event, the contour length of the system increases by the contour length of one domain. Knowing the length of each aminoacid (~0.38 nm) and the number of aminoacids per domain, the inter-peak distance can be estimated and provides an internal control to verify that the peaks correspond to the protein under study. In the case of the I91 domain of titin, the number of amino acids per domain is 89, thus 89 × 0.38 nm - 4 nm = 29.82 nm, very similar to the observed inter-peak distance. The combination of force spectroscopy experiments with molecular dynamics simulations provided a mechanistic description of the unfolding process of titin domains, revealing that particular pairs of β-strands provided the mechanical resistance upon unfolding[17]. Force spectroscopy with AFM has been applied to a number of proteins to study their mechanical properties, in particular to proteins that support forces such as extracellular, cytoskeletal and blood proteins, like filamin, talin and the von Willebrand factor, but also to membrane proteins[20-23]. From these and other studies it seems that β-sheet proteins show important mechanical strength, while α-helical proteins are generally weaker[24].

### Mechanical mapping using AFM

Although pulling, stretching and unfolding proteins using force spectroscopy is a powerful method to probe protein mechanical resistance, the process involves unfolding the protein. Thus, except for proteins that naturally support forces and unfold to undergo function, the forced unfolding process is in general not physiologically relevant for most of the known proteins, and in particular for membrane proteins as they are removed from their native lipid environment. Other methods using AFM have been developed in the recent years that allow obtaining both structural and mechanical information of folded proteins and subdomains and, importantly, of membrane proteins and their surrounding nanoenvironment.

**Figure 5:**
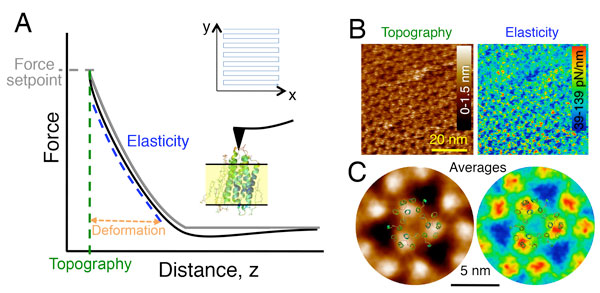
**Mechanical mapping using atomic force microscopy**. A) In mechanical mapping measurements, the tip oscillates in the vertical direction while the tip scans the sample surface (xy plane), generating force versus distance curves at each pixel of the image. In the approaching trace (gray line), the tip approaches the sample until it makes contact (change in slope) reaching the force setpoint, which determines the topography of the sample. If the sample is soft, the tip deforms it as force increases. The tip then retracts back until the initial position is reached (black line). The shape of the curve during contact depends on the contact geometry between the tip and the sample and allows estimating the elasticity of the sample. The sketch represents the setup with the AFM tip deforming the bacteriorhodopsin sample. The actual size of the tip is much larger than the protein. Actually, AFM tips are a few microns high and the radius of the sharp apex of a few nm. B) Topography and elasticity maps of bacteriorhodopsin obtained from mechanical mapping using AFM, revealing the trimeric organization in 2D hexagonal arrays. C) Average images of trimer repeats from B (same colour scale). Overlapped is the top view crystal structure of the bacteriorhodopsin trimer coloured by B-factors. Data replotted from ref. [31] using a tip of ~2 nm radius.

Mechanical mapping (PeakForce™, Quantitative Imaging™ Force volume modes are similar techniques form different commercial AFM systems) is an AFM imaging mode based on force spectroscopy. Mechanical mapping consists of acquiring force versus distance curves at a set compression force while the tip scans the sample in the XY plane (Figure 5A). During scanning, the cantilever repeatedly approaches the surface until the set force is reached and then withdraws back. This results in a force versus distance curve at each pixel of the image. When applying a compression force to a soft material, the sample deforms. The tip indents the sample, and the depth of indentation depends on the shape of the tip, the applied force and the elastic properties of the sample. At a given applied force, the AFM tip will indent more a soft sample than a stiff one. Thus, knowing the applied force and the tip geometry, we can estimate the elastic modulus of the sample from the slope of the contact region (Figure 5A). Topographical, *i.e*. structural, information is obtained from the position at which the setpoint force is reached. This information is provided at each pixel of the image, thus obtaining simultaneous topographical and elasticity maps of the sample surface. The contact geometry between the tip and the sample is a critical issue for a correct estimation of the elastic modulus and different contact elastic models (spherical, pyramidal, conical, cylindrical...) are available depending on the shape of the tip and the range of indentations considered (if the contact between the tip and the sample is well defined, the Young's modulus is the most common used parameter. If the contact is not well defined (such as in high resolution images like Figure 5B) a contact stiffness can be determined instead) [25].

Figure 5B shows an example of mechanical maps of the cytosolic surface of membrane protein bacteriorhodopsin, revealing its characteristic trimeric configuration and hexagonal arrangement found in native membranes. The high-resolution images revealed that interhelical loops are softer, more flexible, than protruding α-helices. This suggested that α-helices provide the mechanical stability to bacteriorhodopsin to maintain their structure, while interhelical loops provide structural flexibility related to conformational changes during the protein photocycle. Interestingly, the elasticity determined using this direct mechanical method is in very good agreement with the neutron scattering measurements described above.

In a recent work, the more complex, native membrane of erythrocytes was also probed using mechanical mapping[26]. The work provides evidence of the mechanical signature of key protein components at the erythrocyte membrane. Thanks to the improved resolution of this technique, the authors show how the metabolic state and assembly of spectrin network and junctional complexes is crucial to preserve the mechanical stability of the membrane. Similar methods using multimodal excitation or torsional cantilevers have been recently developed providing quantitative information about proteins' elasticity[27,28].

Although mechanical mapping is a powerful tool, being a relatively recent technique in their application to individual proteins, and due to the small size of proteins, care has to be taken during experiments. In particular, the sample substrate on which the sample is immobilized may have an effect on the absolute value of the elastic modulus if the indentation depth is too high compared to thin sample thickness [29,30]. Moreover, it is difficult to achieve high-, submolecular resolution on soluble proteins, since the immobilization to the mica surface may partially denature them. Finally, the convolution of the tip size and geometry, difficult to estimate at the nanometer scale may also influence the final results. However, it appears to be one of the only techniques that provide a quantitative, direct determination of mechanical elasticity of individual proteins and submolecular structures in their folded state and native environment.

## Conclusions and future perspectives

As we have seen, the determination of protein flexibility or elasticity is crucial for the understanding of protein function and different complementary techniques for the study are available. Guided by the current tendency towards a more quantitative biology, recent techniques tend to provide absolute quantitative values allowing comparison between different measurements on different proteins. The development of nanotechnologies allows now the manipulation and probing of individual molecules that provide a direct measurement of protein flexibility. In the ideal case, the application of these emerging techniques to the myriad of proteins found in nature will allow in the future the construction of a *mechanome*, a compendium of the mechanical properties of all proteins in an organism in a particular state.

## Competing interests

The authors declare that they have no competing interests.

## Authors' contributions

All authors wrote the paper.

## References

[B1] Laszlo P L'architecture du vivant 2002 Flammarion

[B2] Branden CI Tooze J Introduction to Protein Structure 1999 Garland Pub.

[B3] Whitford D Proteins: Structure and Function 2005 John Wiley & Sons

[B4] Levinthal C Debrunnder JTP, Munck E How to Fold Graciously Mossbauer Spectroscopy in Biological Systems: Proceedings of a meeting held at Allerton House, Monticello, Illinois 1969 University of Illinois Press 22 24

[B5] Frauenfelder H Sligar SG Wolynes PG The Energy Landscapes and Motions of Proteins Science 1991 11 1598 1603 10.1126/science.1749933 1749933

[B6] Ansari A Berendzen J Bowne SF Frauenfelder H Iben IET Sauke TB Shyamsunder E Young RD Protein States and Protein Quakes Proc Natl Acad Sci USA 1985 11 5000 5004 10.1073/pnas.82.15.5000 3860839 PMC390486

[B7] Alberts B Bray D Lewis J Raff M Roberts K Watson JD Molecular Biology of the Cell 1994 3 Garland Publishing, New York

[B8] Yildiz O Vinothkumar KR Goswami P Kuhlbrandt W Structure of the monomeric outer-membrane porin OmpG in the open and closed conformation EMBO J 2006 11 3702 3713 10.1038/sj.emboj.7601237 16888630 PMC1538548

[B9] Zaccai G How Soft Is a Protein? A Protein Dynamics Force Constant Measured by Neutron Scattering Science 2000 11 1604 1607 10.1126/science.288.5471.1604 10834833

[B10] Landau LD Lifshitz EM Theory of Elasticity 1986 Oxford: Pergamon Press

[B11] Johnson KL Contact Mechanics 1985 1 Cambridge: Cambridge University Press

[B12] Lee GU Kidwell DA Colton RJ Sensing Discrete Streptavidin Biotin Interactions with Atomic-Force Microscopy Langmuir 1994 11 354 357 10.1021/la00014a003

[B13] Florin EL Moy VT Gaub HE Adhesion Forces Between Individual Ligand-Receptor Pairs Science 1994 11 415 417 10.1126/science.8153628 8153628

[B14] Moy VT Florin EL Gaub HE Intermolecular Forces and Energies Between Ligands and Receptors Science 1994 11 257 259 10.1126/science.7939660 7939660

[B15] Kellermayer MsSZ Smith SB Granzier HL Bustamante C Folding-Unfolding Transitions in Single Titin Molecules Characterized with Laser Tweezers Science 1997 11 1112 1116 10.1126/science.276.5315.1112 9148805

[B16] Rief M Gautel M Oesterhelt F Fernandez JM Gaub HE Reversible unfolding of individual titin immunoglobulin domains by AFM Science 1997 11 1109 1112 10.1126/science.276.5315.1109 9148804

[B17] Marszalek PE Lu H Li H Carrion-Vazquez M Oberhauser AF Schulten K Fernandez JM Mechanical unfolding intermediates in titin modules Nature 1999 11 100 103 10.1038/47083 10573426

[B18] Evans E Ritchie K Dynamic strength of molecular adhesion bonds Biophys J 1997 11 1541 1555 10.1016/S0006-3495(97)78802-7 9083660 PMC1184350

[B19] Bustamante C Marko JF Siggia ED Smith S Entropic Elasticity of Lambda-Phage Dna Science 1994 11 1599 1600 10.1126/science.8079175 8079175

[B20] Schwaiger I Kardinal A Schleicher M Noegel AA Rief M A mechanical unfolding intermediate in an actin-crosslinking protein Nat Struct Mol Biol 2004 11 81 85 10.1038/nsmb705 14718927

[B21] del Rio A Perez-Jimenez R Liu R Roca-Cusachs P Fernandez JM Sheetz MP Stretching Single Talin Rod Molecules Activates Vinculin Binding Science 2009 11 638 641 10.1126/science.1162912 19179532 PMC9339221

[B22] Zhang X Halvorsen K Zhang CZ Wong WP Springer TA Mechanoenzymatic Cleavage of the Ultralarge Vascular Protein von Willebrand Factor Science 2009 11 1330 1334 10.1126/science.1170905 19498171 PMC2753189

[B23] Oesterhelt F Oesterhelt D Pfeiffer M Engel A Gaub HE ller DJ Unfolding Pathways of Individual Bacteriorhodopsins Science 2000 11 143 146 10.1126/science.288.5463.143 10753119

[B24] Crampton N Brockwell DJ Unravelling the design principles for single protein mechanical strength Curr Opin Struct Biol 2010 11 508 517 10.1016/j.sbi.2010.05.005 20542682

[B25] Rico F Roca-Cusachs P Gavara N Farre R Rotger M Navajas D Probing mechanical properties of living cells by atomic force microscopy with blunted pyramidal cantilever tips Phys Rev E Stat Nonlin Soft Matter Phys 2005 11 021914 16196611 10.1103/PhysRevE.72.021914

[B26] Picas L Rico F Deforet M Scheuring S Structural and Mechanical Heterogeneity of the Erythrocyte Membrane Reveals Hallmarks of Membrane Stability ACS Nano 2013 10.1021/nn303824j 23347043

[B27] Dong M Husale S Sahin O Determination of protein structural flexibility by microsecond force spectroscopy Nat Nanotechnol 2009 11 514 517 10.1038/nnano.2009.156 19662014

[B28] Martinez-Martin D Herruzo ET Dietz C Gomez-Herrero J Garcia R Noninvasive Protein Structural Flexibility Mapping by Bimodal Dynamic Force Microscopy Phys Rev Lett 2011 11 198101 21668203 10.1103/PhysRevLett.106.198101

[B29] Picas L Rico F Scheuring S Direct Measurement of the Mechanical Properties of Lipid Phases in Supported Bilayers Biophys J 2012 11 L01 L03 10.1016/j.bpj.2011.11.4001 22225813 PMC3250677

[B30] Gavara N Chadwick RS Determination of the elastic moduli of thin samples and adherent cells using conical atomic force microscope tips Nat Nano 2012 advance online publication 10.1038/nnano.2012.163 PMC3492504 23023646

[B31] Rico F Su C Scheuring S Mechanical Mapping of Single Membrane Proteins at Submolecular Resolution Nano Lett 2011 11 3983 3986 10.1021/nl202351t 21800925

